# Bioluminescence resonance energy transfer-based biosensors allow monitoring of ligand- and transducer-mediated GPCR conformational changes

**DOI:** 10.1038/s42003-018-0101-z

**Published:** 2018-08-07

**Authors:** Louis-Philippe Picard, Anne Marie Schönegge, Martin J. Lohse, Michel Bouvier

**Affiliations:** 1 Department of Biochemistry and Molecular Medicine, Institute for Research in Immunology and Cancer, Université de Montreal, Montreal, QC H3C 3J7 Canada; 2 Institute of Pharmacology and Toxicology, 97078 Würzburg, Germany; 3 Present Address: Max Delbrück Center for Molecular Medicine, 13125 Berlin, Germany

## Abstract

G protein-coupled receptors (GPCRs) are seven-transmembrane proteins that mediate a variety of cellular response which make them a target of choice for drug development in many indications. It is now well established that GPCRs can adopt several distinct conformations that can be differentially stabilized by various ligands resulting in different biological outcomes, a concept known as functional selectivity. However, due to the highly hydrophobic nature of GPCRs, tools to monitor these conformational ensembles are limited and addressing their conformation dynamics remains a challenge with current structural biology approaches. Here we describe new bioluminescent resonance energy transfer-based biosensors that can probe the conformational rearrangement promoted by ligands with different signaling efficacies as well as the impact of transducers such as G proteins and β-arrestin on these conformational transitions. The design of such sensors for other receptors should be useful to further explore the structural determinants of GPCR functional selectivity.

## Abstract (web summary)

Louis-Philippe Picard et al. present a BRET-based biosensor for monitoring ligand- and transducer-mediated conformational changes in GPCRs. They apply the biosensor to β2AR, a prototypical class A GPCR, and demonstrate the ability of the biosensor to be multiplexed with other BRET-based biosensors.

## Introduction

G protein-coupled receptors (GPCRs) form the largest family of membrane proteins involved in signal transduction, play central roles in multiple biological processes, and, as such, are the target for the development of drugs in many clinical indications. In their classical representation, GPCRs promote their cellular effect by the engagement and activation of selective G proteins, while the engagement of β-arrestin leads to desensitization and internalization^1^. However, in recent years it has been clearly established that individual receptors can engage multiple G protein subtypes and that in addition to its role in desensitization, β-arrestin also leads to intrinsic signaling activity including mitogen-activated protein kinase activation^2,3^. Recently, the observation that each GPCR can engage multiple signaling pathways^4,5^ coupled to the concepts of functional selectivity and ligand-biased signaling^6–8^ have raised the possibility of identifying ligands that selectively modulate the therapeutically relevant pathways while avoiding those responsible for undesirable side effects^9,10^. It has been proposed that such ligand-biased signaling results from the stabilization of different receptor conformation ensembles that select distinct signaling partners, such as G proteins or β-arrestin^11–14^. For the β2-adrenergic receptor (β2AR), compounds such as salbutamol (SALB) and salmeterol (SALM) have been shown to be efficacious partial agonist for the stimulatory G protein (Gs), while poorly promoting the recruitment of β-arrestin^15^. However, monitoring these ligand-specific conformations remains a challenge, in particular when considering the allosteric nature of the receptor’s interaction with cellular tranducers in their native cellular environment. Recently, fluorescent resonance energy transfer (FRET)^16^ and fluorescein arsenical hairpin binder-FRET (FlAsH-FRET)^17–19^ probes have been introduced in GPCR constructs to monitor the intramolecular conformational changes promoted by ligands with different efficacies. When compared to FRET, bioluminescent resonance energy transfer (BRET)-based sensors, such as those developed herein, present several advantages. Notably, because there is no direct activation with light, no artifactual direct excitation of the RET acceptor can occur, thus limiting the background. For the same reason, autofluorescence or photobleaching that can limit FRET applications is not an issue with BRET. A direct comparison of conformational BRET and FRET-based sensors is presented in an accompanying paper^20^. FlAsH-BRET has been used to probe conformational rearrangements^18,19^. Although it has the advantage of having an energy acceptor that is smaller than a fluorescent protein, it requires exogenous labeling and extensive washing, which make the assay less convenient. None of these studies assessed the impact of transducers' engagement on the conformational ensembles of the receptors. Probing transducers’ influence on receptor conformations is of particular interest when considering the major differences observed between the agonist-bound β2AR conformations in the presence or absence of Gs^21,22^. Indeed, directly monitoring the dynamics of the conformation ensembles resulting from the engagement of the receptor by both ligands and transducers should prove useful to understand how drugs can selectively promote the engagement of subsets of their downstream transducers.

In the present study, taking advantage of the Oplophorus Gracilirostris-derived luciferase (Nluc) brightness^23^, we developed a BRET-based biosensor that can be multiplexed with other BRET-based assays to monitor receptor conformational changes and the engagement of cellular transducers in parallel in living cells.

## Results

### Biosensors design and characterization

**Fig. 1:**
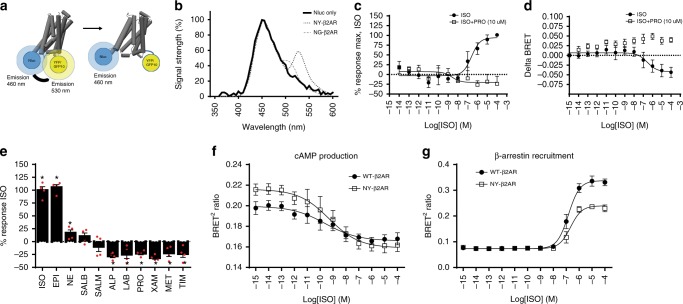
Description and functional characterization of the intramolecular β2AR conformational biosensors. **a** Schematic representation of the biosensors. **b** Bioluminescence emission spectra of the YFP (NY-β2AR) and GFP10 (NG-β2AR) versions of the biosensor. **c** Concentration–response curves of the NY-β2AR conformational biosensor following isoproterenol (ISO) stimulation, in the presence or absence of 10 μM propranolol (PRO). The data are express as % of the maximal ISO-promoted response. **d** Absolute ΔBRET values of a typical experiment. **e** BRET changes in the NY-β2AR induced by ligands with different intrinsic efficacy. Statistical analysis was performed using Student’s *t* tests with Holm–Sidak correction for multiple comparison (*p value <0.05). **f**, **g** Concentration–response curves for cAMP production (**f**) and recruitment of β-arrestin (**g**), using GFP10-mut-EPAC1-RlucII (a decrease in BRET signal indicates an increase in cAMP production) and rGFP-CAAX/β-arrestin2-RlucII (an increase in BRET indicates a recruitment of β-arrestin to the receptor) BRET sensors, respectively, upon activation of NY-β2AR or the Flag-tagged wild-type β2AR (WT-β2AR) (for equivalent receptor levels; see Supplementary Fig. 2). In all cases, data are expressed as the mean ± SEM from three to five independent experiments conducted in duplicates

The β2AR, a prototypical class A GPCR was used as a study model since the structures of both inactive and active conformations have been solved. Furthermore, multiple signaling pathways have been characterized for this receptor and several biased ligands are available. To probe the movement associated with receptor conformational rearrangements, Nluc, a luciferase which is brighter and smaller than the traditionally used renilla luciferase (Rluc), was used as the BRET energy donor. Nluc was introduced in the third intracellular loop (ICL3; between positions 251 and 252) of the receptor. The archetypal BRET1^24,25^ or BRET2^26,27^acceptors, YFP or GFP10, were fused to the C terminus of the receptor (position 369) (see Methods and Fig. 1a). These positions detect the rotation of TM5 and the outward movement of TM6 that bring ICL3 away from the C-termini^16^.

Two different energy acceptors, YFP (NY-β2AR) and GFP10 (NG-β2AR) (NY and NG stand for Nluc-YFP or Nluc-GFP10, BRET pairs), were tested. The spectra of the two BRET pairs were obtained using coelenterazine 400a (Coel400a) as the Nluc substrate. As shown in Fig. 1b, the YFP construct yielded a more efficient transfer (60% vs. 45% for YFP and GFP10, respectively) and a better separation between the donor and acceptor emission peaks (78 nm for YFP vs. 50 nm for GFP10).

The NY-β2AR biosensor was then used to probe the activation-induced conformational changes promoted by the full agonist isoproterenol (ISO) in the presence or absence of the antagonist propranolol (PRO) (Fig. 1c, d). The concentration-dependent ISO-promoted decrease in BRET was completely blocked by the addition of PRO, demonstrating that the biosensor detected conformational changes associated with activation that are consistent with a separation between the C terminus and ICL3, which is observed in the active conformation of the receptor^16,28^. To determine whether the biosensor can detect the distinct conformation ensembles stabilized by ligands with different intrinsic efficacy, the effect of agonists, partial agonists, and inverse agonists was tested. The different changes in BRET signal detected for these different ligands (Fig. 1e) correlated well with the known efficacy of the compounds for Gαs activation, consistent with the notion that different groups of ligands stabilize distinct conformational ensembles.

### Biosensor functionality and multiplexing

To assess the possible impact of the BRET probes on the functionality of the NY-β2AR conformation sensor, its ability to promote cAMP production and β-arrestin recruitment was compared to that of the wild-type β2AR. Taking advantage of the substrates specificity between Nluc and RlucII (with crossover of <3%; Supplementary Fig. 1), Coel400a was used to monitor the signal from Nluc and methoxy-e-coelentrazine from RlucII. As seen in Fig. 1f, g, at similar expression levels of NY-β2AR and wild-type β2AR (Supplementary Fig. 2), both receptor constructs resulted in cAMP production and β-arrestin recruitment, detected by BRET using RlucII-EPAC-GFP10^29^ and β-arrestin-RlucII/rGFP-CAAX^30^ biosensors, respectively. Although the extent of β-arrestin recruitment detected for NY-β2AR is somewhat smaller than the one observed for wild-type β2AR, the biosensor is functional and can transduce signals.

**Fig. 2:**
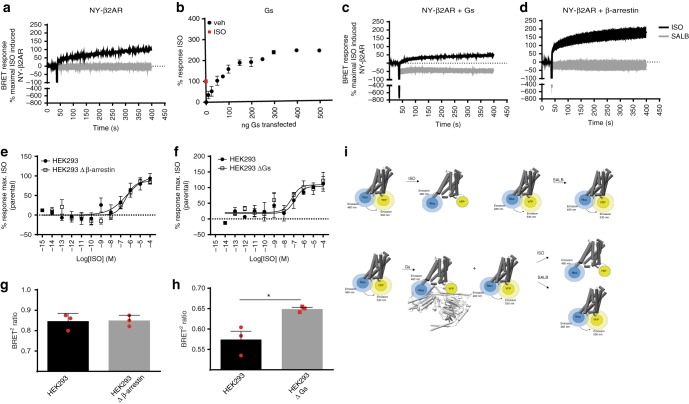
Effects of Gs and β-arrestin on conformational changes promoted by isoproterenol (ISO) and salbutamol (SALB). **a** Kinetics of the changes in BRET signal of the NY-β2AR conformational sensor upon ISO and SALB (100 μM) stimulation in HEK293T cells. Data are expressed as normalized ligand-promoted BRET changes; the maximal response of the biosensor in response to ISO being set as 100%. Although the ISO-promoted conformational change results in a decrease in BRET, the BRET changes are represented as positive responses. **b** Gαs-promoted change in the NY-β2AR conformational sensor BRET signal as a function of the amount of Gαs co-transfected. Data are expressed as the percentage of ISO response in the absence of over-expressed G protein. The response to ISO in the absence of over-expressed Gs is represented by the square symbol on the *Y*-axis. **c**, **d** Effect of co-transfection of Gs or β-arrestin (500 ng per 10^6^ cells) on the kinetics of ISO-promoted and SALB-promoted conformational changes of the NY-β2AR sensor. Data are expressed as the percentage of ISO-promoted response in the absence of Gs and β-arrestin over-expression (**a**), the dotted line corresponding to the basal BRET signal observed in the presence of over-expressed Gs or β-arrestin in the absence of ligand. **e**, **f** Concentration–response curves of the ISO-stimulated NY-β2AR response in HEK293 cells lacking β-arrestin vs. their parental cells (**e**) or in HEK293 cells lacking Gs vs. their parental cells (**f**). **g**, **h** Basal BRET levels observed in the different cell lines. Statistical analysis was performed using an unpaired Student’s *t* test (*p value <0.05). Data are expressed as percentage of ISO response for the parental cell lines and represent the mean ± SEM of at least three independent experiments conducted in duplicates. **i** Schematic cartoon of ISO-induced vs. SALB-induced conformational changes

Using a similar experimental design, we multiplexed the detection of the BRET-based sensors to evaluate in parallel Gαs activation, cAMP production, or β-arrestin engagement on the one hand and the receptor conformational changes on the other. As shown in Supplementary Fig 3, the multiplexing mode shows that the concentration-dependent ISO-promoted conformational change detected by NY-β2AR is accompanied by increases in Gs activation, cAMP accumulation, and β-arrestin recruitment. The potency of ISO to promote the conformational change (negative logarithm of the EC50 (pEC_50_): −7.2, −6.9, and −7.2 in the presence of the Gs, cAMP, and β-arrestin biosensors, respectively) was well correlated with its potency to promote Gs activation (pEC_50_: −7.2) and β-arrestin engagement (pEC_50_: −7.3). The amplification between the Gs activation and the cAMP is clearly seen by the left shift in the potency of ISO to stimulate cAMP production (pEC50: −8.9). In contrast, SALB, which is a biased ligand^15^ activating Gs (albeit to a lower extent than ISO) but only marginally promoting the engagement of β-arrestin, did not induce any conformational changes detectable with NY-β2AR, even at a concentration maximally occupying the receptor. This difference between ISO-promoted and SALB-promoted conformational changes was also observed in kinetic experiments (Fig. 2a). The observation that SALB was equally efficacious to ISO in promoting cAMP production (Supplementary Fig. 3d) suggests that the difference in the ability of the two ligands to promote the receptor conformational change did not result from a difference in protein kinase A-mediated phosphorylation of the receptor.

Control experiments confirmed that cross-contamination between the BRET configurations in the multiplexing experiments did not adversely affect the data. Indeed, as shown in Supplementary Fig. 1, no important contribution of the possible transfer from Nluc to GFP10 to the signal detected for the Nluc transfer to YFP occurred in the multiplexed configurations. Similarly, the possible transfer of Rluc to YFP did not contribute to the signal detected for the Rluc transfer to GFP10 in the NY-β2AR/Gs (Gαs117RlucII/Gγ1-GFP10) multiplexed configuration. However, the transfer of energy between the Rluc of the GFP10-linker-RlucII-pβarr2 sensor and the YFP of NY-β2AR contributed to the BRET signal detected for the ISO-promoted recruitment of β-arrestin2 to the NY-β2AR in the multiplexed configuration. This resulted in an amplification of the β-arrestin2 engagement signal observed with NY-β2AR compared to wild-type β2AR (Supplementary Fig 1g). This phenomenon can be advantageously used to increase the signal window for a given sensor. However, it highlights the fact that the possible contamination of one acceptor signal by the other should always be controlled for and taken into consideration when multiplexing BRET sensors and selecting the configuration of the assays.

### Evaluation of transducers' effect on the conformational sensor

The above results suggest that different conformation ensembles are stabilized by ISO and SALB and raise the possibility that the engagement of Gs and/or β-arrestin may contribute to the conformational changes detected. To test this hypothesis, we evaluated the impact of G proteins and β-arrestin over-expression on the conformational change detected. As shown in Fig. 2b, over-expression of Gs led to significant concentration-dependent conformational changes of NY-β2AR, indicating that coupling to Gs in the absence of agonist is sufficient to induce such changes. The effect of Gs was selective, since over-expression of G12 or Gi had much weaker effects on the conformation (Supplementary Fig. 4). Such ligand-independent conformational changes imposed by the G proteins is consistent with the notion that some receptors may be precoupled to G proteins^31–33^. However, we cannot exclude the possibility that such precoupling is forced by over-expressing the G proteins.

In the presence of over-expressed Gs, stimulation with ISO promoted additional BRET changes, but to a lower extent than in the absence of over-expressed Gs (Fig. 2a, c). Whether the agonist promotes further changes in receptor conformation or stabilizes a larger fraction of the receptors coupling to Gs, remain to be established. However, the fact that the BRET change promoted by Gs (Fig. 2b) is hyperbolic and reaches saturation supports the former hypothesis. Over-expression of β-arrestin on its own did not affect the conformation of NY-β2AR (Supplementary Fig. 4). However, it potentiated the ISO-promoted BRET change (Fig. 2d), suggesting that β-arrestin modifies the agonist-bound receptor conformational ensembles. This could result either from a larger conformational change promoted by β-arrestin than by Gs or from the stabilization of a greater proportion of the receptor in the agonist-promoted conformation.

The observation that both Gs and β-arrestin modulate the receptor’s conformation ensemble detected by NY-β2AR could suggest that the change is entirely due to the transducer binding. To determine if this is the case or if ISO can promote a conformational change on its own, the conformational change was assessed in cells devoid of Gs or β-arrestin. For this purpose, we took advantage of the Gs and β-arrestin-deficient cell lines that were recently generated using CRISPR/Cas9^34,35^. The lack of Gs and β-arrestin was functionally confirmed by the absence of ISO-stimulated cAMP production and β2AR endocytosis in the Gs and β-arrestin-deficient cells, respectively, when compared with their parental cells (Supplementary Fig. 5). As shown in Fig. 2e, f, the lack of either transducer did not prevent the ISO-promoted conformational changes of the receptor, but the basal BRET observed for the NY-β2AR sensor was higher in the cells lacking functional Gs (Fig. 2h), indicating that native expression levels of Gs may be sufficient to induce a conformational change in NY-β2AR, yielding a reduction of BRET consistent with the effect observed in the presence of over-expressed Gs (Fig. 2b). In cells lacking β-arrestin, treatment with cholera toxin to eliminate the contribution of receptor-bound Gs (cholera toxin leads to a constitutive activation and long term down-regulation of Gs^36^), ISO still promoted NY-β2AR BRET change (Supplementary Fig. 6), indicating that ISO binding on its own is sufficient to change the conformation ensembles of the receptor.

In contrast to ISO, SALB did not promote any significant change in the BRET signal of NY-β2AR for the time period examined (Figs, 1e and 2a). Not surprisingly, over-expression of β-arrestin did not influence this lack of response. However, a rapid and sustained decrease in the Gs-promoted conformational response was observed upon SALB stimulation (Fig. 2c). This decrease was not observed in the concentration–response curves presented in Supplementary Fig. 3, most likely due to the lower level of Gs expressed in these multiplexing experiments that was not sufficient to significantly affect the conformational ensemble of NY-β2AR. The fact that SALB reduced the conformational change resulting from the constitutive Gs coupling upon over-expression indicates that the partial agonist changed the equilibrium between Gs-bound and Gs-free receptor. Such reduction in the proportion of receptor in the Gs-induced conformation cannot be compensated by the recruitment of β-arrestin, since SALB does not promote efficient recruitment of β-arrestin (Supplementary Fig. 3). Taken together, these data clearly indicate that the biased partial agonist, SALB, while activating Gs, promotes conformational rearrangements that are clearly distinct from that of ISO and that these conformations are differentially influenced by the receptor’s transducers (Fig. 2i). Consistent with the notion that the stabilization of distinct conformational ensembles is a characteristic of the biased and partial agonist nature of SALB, another partial and Gs-biased ligand, SALM, failed to promote detectable conformational change on its own and also reduced the Gs-promoted conformational change of the receptor (Supplementary Fig. 7).

## Discussion

In summary, we have developed a conformational biosensor for the β2AR that can distinguish between compounds with distinct signaling efficacies in living cells, as well as monitor the impact of transducers on receptor conformation. Such biosensors can be easily combined with other BRET-based sensors to correlate conformational rearrangements with signaling profiles. The generalizability of such biosensors is illustrated in an accompanying paper^20^ where similar biosensors were created for the α1AR and parathyroid hormone-related protein receptor in addition to β2AR. Even though the approach is generalizable, special attention should be given to the design of the biosensors. In particular, the position in which the biosensor components (energy donors and acceptors) are introduced should be selected with care. Indeed, the insertion of relatively large inserts in the receptor structure could have detrimental effects on the signaling activity of some receptors. Controls experiments to asses such potential impact are therefore required and any alterations in the signaling profile observed should be taken into consideration in the interpretations of the data.

The observation that Gs significantly increased the ISO-promoted decrease in BRET signal observed with the NY-β2AR sensor is in agreement with the previously published study using single-molecule FRET^37^ between the TM4 and TM6 in which an amplification of the ligand-induced changes in conformation promoted by epinephrine (EPI) was observed in the presence of Gs. Such amplification of the conformational changes in the presence of Gs is consistent with the crystal structures obtained for the agonist-bound β2AR^28,38^. Indeed, the opening of the TM6 away from the core of the receptor to create a cradle for the C-tail of the G protein α-subunit is much larger in the crystals obtain for the agonist-bound receptor in complex either with Gαs^28^ or a nanobody mimicking the α-subunit of Gs^38^. It follows that the NY-β2AR conformation sensor can be used to probe the allosteric conformational changes promoted by both ligands and transducers in living cells.

In the case of β-arrestin, its over-expression alone does not promote any detectable conformation change. This lack of effect is expected when considering the low constitutive activity of this pathway for the β2AR. The results of the over-expression of β-arrestin upon ligand stimulation demonstrate the difference in activation mechanism of biased ligands such SALB and SALM in comparison to ISO. In the case of ISO stimulation, the over-expression of β-arrestin amplified the BRET changes detected by the conformation sensor, whereas in the case of SALB and SALM no response was observed. This is in keeping with the fact that the biased ligands SALB and SALM are poor recruiter of β-arrestin; therefore, β-arrestin cannot stabilize the ligand-induced response such as that observed with ISO. These experiments demonstrate the utility of the conformation sensor in living cells to better probe the structural determinants underlying functional selectivity.

In addition, to probe the activation of the receptors with known ligands and transducers, the BRET-based GPCR conformational sensors should prove useful to probe both ligand and transducer-promoted conformational changes to identify ligands for orphan receptors as well as identifying the transducers coupled to a given receptor. Finally, the sensors could be used to monitor the effect of mutations on the conformation changes of GPCRs upon activation by ligands with different biases to further explore the specific residues and receptor domains involved in ligand-biased signaling.

## Methods

### Reagents

(−)-Isoproterenol hydrochloride, (−)-epinephrine (EPI), (−)-norepinephrine (NE), alprenolol hydrochloride (ALP), labetalol hydrochloride (LAB), (±)-propranolol hydrochloride (PRO), metoprolol tartrate (MET), timolol maleate (TIM), and cholera toxin (CTX) were purchased from Sigma-Aldrich. Salbutamol hemisulfate (SALB) and xamoterol hemifumarate (XAM) were purchased from Tocris Bioscience. Salmeterol xinofoate (SALM) was purchased from Selleckchem. Coelenterazine 400a and methoxy-e-coelenterazine were purchased from NanoLight Technology.

### Plasmids

The GFP10-mutEPAC1-RlucII^29^, GFP10-linker-RlucII-pβarr2^39^, Flag-β2AR^40^, Gαs-117-RlucII^40^, Gβ1^41^, Gγ1-GFP10^41^, β-arrestin2-RluII^42^, and rGFP-CAAX^30^ were previously described. The Gαs, Gαi2, Gα12, Gγ1, and hβ-arrestin2 plasmids were purchased from cDNA.org. The pNL1.1 plasmid was purchased from Promega. The NY-β2AR sensor was obtained by Gibson assembly, using the previously published flag-β2AR FRET sensor^16^, and replacing the CFP by a Nluc. The YFP was then replaced by a GFP10 to obtain the NG-β2AR version of the sensor.

### Cell culture and transfection

HEK293T is the cell line in which BRET-based biosensors have been developed in Dr. Bouvier’s laboratory and this cell line was used for all the BRET and enzyme-linked immunosorbent (ELISA) experiments. HEK293T cells were grown in Dulbecco’s modified Eagle’s medium (DMEM) supplemented with 10% newborn calf serum at 37 °C with 5% CO_2_. HEK293-ΔGs and HEK293-Δβarr1/2 were generated by CRISPR/Cas9 gene editing as previously reported^34,35^. HEK293-ΔGs, HEK293-Δβarr1/2, and their respective parental cells were grown in DMEM supplemented with 10% fetal bovine serum at 37 °C with 5% CO_2_. For transfection, cells were detached with trypsin, diluted at a concentration of 500,000 cells per mL, and transfected with 2.5 μg of total DNA for 10^6^ cells using linear polyethylenimine (PEI, Polysciences) as transfecting agent with a PEI:DNA ratio of 3:1. Directly after transfection, cells were plated in white 96-well culture plates (Greiner) coated with poly-l-ornithine (Sigma-Aldrich) at a concentration of 50,000 cells per well and were incubated for 48 h before the experiment. Cells were regularly tested for mycoplasma contamination (PCR Mycoplasma Detection Kit, abm).

### Bioluminescence spectral profiles

HEK293T cells were transfected with the indicated constructs as described above. The luminescence spectra between 360 and 600 nm were acquired with steps of 5 nm, immediately after the addition of 2.5 μM of Coel400a using a FlexStationII microplate reader (Molecular Devices). The bioluminescence is expressed as a percentage of the maximal emission.

### BRET measurements

Forty-eight hours after transfection, cells were washed with stimulation buffer (Hank’s balanced salt solution, HBSS). For the conformational sensor alone (NY-β2AR), Coel400a, diluted in stimulation buffer, was added (2.5 μM final) for 6 min. Increasing concentrations of ISO, diluted in a stimulation buffer, or 10 μM of different ligands were then added for 5 min. BRET was monitored with a TriSTAR2 LB 942 microplate reader (Berthold Technologies) equipped with a donor filter 485/20 nm and an acceptor filter 530/25 nm. For the multiplexing, the conformational biosensor (NY-β2AR) was co-transfected with the individual transducer biosensors. The response of the NY-β2AR was measured as described above. The transducer biosensors were measured in separate wells. Methozy-e-coelenterazine (0.25 μM) was added for 6 min, followed by incubation with increasing concentrations of ISO, in stimulation buffer, for 5 min (Gs) or 15 min (cAMP production and β-arrestin recruitment). BRET was then monitored with a TriSTAR2 LB 942 microplate reader (Berthold Technologies) equipped with a donor filter of 410/80 nm and an acceptor filter of 515/40 nm. In all cases, BRET ratio was calculated by dividing the acceptor emission over the donor emission.

### Conformational sensor

HEK293T, HEK293-ΔGs, HEK293-Δβ-arrestin, and their respective parental cells were either transfected with the YFP (NY-β2AR) version of the conformational biosensor alone or in combination with specific sensors for different pathways (see below), or in combination with different G proteins (Gαs, Gα12 or Gαi2) or β-arrestin2. BRET was then monitored as described above.

### cAMP production

HEK293T cells were co-transfected with the conformational biosensor (NY-β2AR) or the wild-type β2AR and the BRET-based biosensor GFP10-mutEPAC1-RlucII^29^. BRET was then monitored as described above. The conformational change of the GFP10-mutEPAC1-RlucII after cAMP binding leads to a decrease in the BRET ratio.

### β-Arrestin recruitment

HEK293T cells were co-transfected with the conformational biosensor (NY-β2AR) or the wild-type β2AR and the BRET-based plasma membrane translocation biosensors rGFP-CAAX/βarr2-RlucII^30^. BRET was then monitored as described above. Recruitment of β-arrestin to the receptor induces a change in localization of the β-arrestin to the plasma membrane that leads to an increase in BRET ratio.

### β-Arrestin engagement

HEK293T cells were co-transfected with the conformational biosensor (NY-β2AR) or the wild-type β2AR and the plasma membrane anchored BRET-based biosensor GFP10-linker-RlucII-pβarr2^39^. BRET was then monitored as described above. The recruitment of β-arrestin to the stimulated receptor increases the proximity between RlucII and GFP10, leading to an increase in BRET signal.

### Gs activation

HEK293T, HEK293-Δβ-arrestin, or their parental cells were co-transfected with the conformational biosensor (NY-β2AR) and a three-component BRET-based biosensor, Gαs117RlucII, Gβ1, and Gγ1-GFP10^40^. BRET was then monitored as described above. The dissociation of the Gα and Gβ/Gγ subunits after activation leads to a decrease in the BRET ratio.

### Endocytosis

HEK293-Δβ-arrestin and their parental cells were co-transfected with the β2AR-RlucII construct and the FYVE-rGFP biosensor. BRET was then monitored as described above. The translocation of the β2AR-RlucII from the membrane to the early endosomes (marked with the sensor FYVE-rGFP) leads to an increase in the BRET signal.

### Kinetics

HEK293T cells were transfected with the YFP version of the conformational biosensor (NY-β2AR) alone or in combination with trimeric Gs or β-arrestin2. Cells were washed with stimulation buffer, HBSS. BRET was monitored 5 min after the addition of 2.5 μM of Coel400a, at every 0.72 s, for a total time of 6 min with an injection of vehicle, 100 μM ISO, or 100 μM SALB at the 40 s time point. The first 40 s represent the basal state of the biosensor and the mean of these time points was used as 0%, while the last time point stimulated with ISO without co-transfection of Gs or β-arrestin represents 100% of the response. The reading was done on a Mithras LB 940 microplate reader (Berthold Technologies) equipped with a donor filter of 485/20 nm and an acceptor filter of 530/25 nm.

### Total fluorescence measurements

HEK293T cells were transfected with NG-β2AR and GFP10-mutEPAC1-RlucII, as described above. The total fluorescence was monitored using a FlexStationII microplate reader with a combination of excitation at 400 nm and emission at 510 nm for GPF10, and with excitation at 485 nm and emission at 538 nm for YFP.

### Luciferase measurements

Luminescence of NG-β2AR and GFP10-mutEPAC1-RlucII sensors was monitored 5 min after the addition of 2.5 μM Coel400a or 0.25 μM methoxy-e-coelenterazine, with a TriSTAR2 LB 942 microplate reader equipped with 485/20 and 410/80 nm, respectively, corresponding to the filters used for the Nluc and RlucII in BRET experiments. The relative expression of each biosensors was monitored by measuring the fluorescence of the GFP10 of NG-β2AR or GFP10-mutEPAC1-RlucII sensors excited at 410/8 nm.

### Cell surface ELISA

HEK293T cells were transfected with the Flag-tagged NG-β2AR and wild-type β2AR constructs, as described above. Cells were washed two times with phosphate-buffered saline (PBS), and then fixed with 3% paraformaldehyde diluted in PBS for 15 min. Fixed cells were washed three times with WashB solution (0.5% bovine serum albumin in PBS). The primary antibody (anti-FLAG M2 from Sigma-Aldrich) was added at a dilution of 1:10,000, and cells were incubated for 1 h at 25 °C. After the incubation, cells were washed three times with WashB solution. The horse radish peroxidase-conjugated secondary antibody against mouse IgG (GE healthcare) was added at a dilution of 1:1000 and cells were incubated for 1 h at 25 °C. After the incubation, cells were washed three times with WashB solution. Fifty microliters of HBSS was added per well, and 2 min before the reading, 50 μL of ECL (Perkin Elmer) was added. Total bioluminescence was monitored with a TriSTAR2 LB 942 microplate reader.

### Data analysis

All data were analyzed using GraphPad PRISM (GraphPad Software, La Jolla, CA, USA). A four-parameter non-linear logistic equation was used to analyze the concentration–response curves, whereas unpaired or multiple *t* test analysis was used to evaluate the statistical difference of single-concentration experiments. All data are represented by the mean ± SEM of multiple independent experiments.

### Data availability

The authors declare that all data supporting the findings in this study are presented within the article and its Supplementary Information Files and are available from the corresponding author upon request.

## Electronic supplementary material

Supplementary Information
