# Supplementary material for: Bioluminescence resonance energy transfer-based biosensors allow monitoring of ligand- and transducer-mediated GPCR conformational changes
Source: Commun Biol. 2018 Aug 7;1:106. doi: 10.1038/s42003-018-0101-z (PMC6123734; doi:10.1038/s42003-018-0101-z)
Supplement: Supplementary file 1 — Supplementary Information [file 42003_2018_101_MOESM1_ESM.pdf]

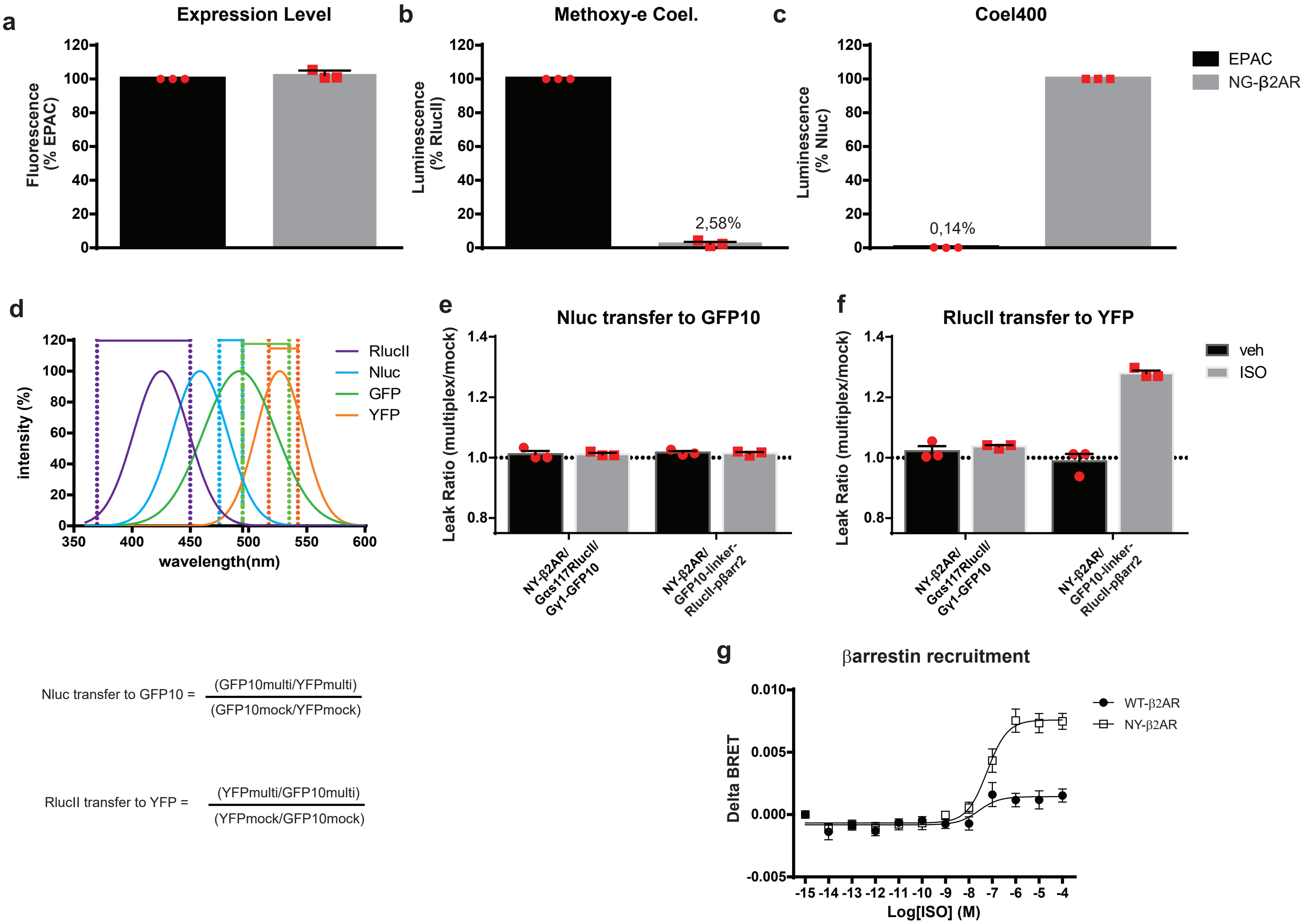

Supplementary Figure 1. Comparison of NLuc (NY-β2AR and NG-β2AR) and Rluc (EPAC) luminescence emission in the presence of their appropriate substrate. a) Total fluorescence of Rluc-EPAC-GFP10 and NG-β2AR expressed as a percentage of the EPAC signal. b-c) Luminescence in cells expressing either Rluc-EPAC-GFP10 or NG-β2AR was measured 5 minutes after the addition of methoxy-e-coelenterazine(b) or coelenterazine 400a(c). Data are expressed as a percentage of the luminescence emitted in the channel corresponding to the wavelengths associated with each of the substrates (400 nm for methoxy-e-coelenterazine and 480 nm for Coel-400). d) Theoretical emission spectra of RlucII, Nluc, GFP10, and YFP with the passing bands of the filters used for their detection (dotted rectangles). e-f) Evaluation of the transfer from Nluc to GFP10 (e) and from RlucII to YFP (f) in the multiplexing experiments to assess the possible contaminating emission of GFP10 and YFP upon excitation by Nluc/Coel-400 and Rluc/methoxy-e-coelenterazine, respectively. The transfer from Nluc to GFP10 was monitored by measuring the ratio of the emission in the GFP10 channel (515/40 nm) over the emission in the YFP channel (530/25 nm) after the addition of Coel-400, in the presence or absence of ISO. The transfer from RlucII to YFP was monitored by measuring the ratio of the emission in the YFP channel (530/25 nm) over the emission in the GFP channel (515/40 nm) after addition of methoxy-e-coelenterazine, in the presence or absence of ISO. This ratio in the multiplex condition was divided by the one obtained in a mock condition were the second sensor was not present. The equation used to assess the cross contamination between the multiplexed signals is shown at the bottom of panel (d). A ratio of 1 represents no detectable cross-transfer. g) Concentration-response curves for ISO stimulation of NY-β2AR sensor compared with WT-β2AR for β-arrestin recruitment assessed using the GFP10-linker-RlucII-pβarr2 sensor. Results show an increased signal for the NY-β2AR sensor compared with the WT due to the transfer of the RlucII to the YFP. Data represent the mean ± SEM of 3 independent experiments conducted in duplicates. The red dots and squares in a,b,c,e and f indicate the individual data points obtained in each of the independent experiments. Overall, the results show that essentially no cross-contaminating emission of the Nluc emission into the Rluc channel (less than 1%) or reciprocally of the Rluc emission in the Nluc channel (less than 3%) was detected when Coel-400 and methoxy-e-coelenterazine were used respectively as substrates (b-c). The data also show that possible contamination transfer from Nluc to GFP10 does not contribute to the signal detected for the Nluc transfer to YFP in the multiplexed configurations (ratios between 1.01 and 1.02 for the various conditions(e)). Similarly, no significant contribution of the transfer of Rluc to YFP to the signal detected for Rluc transfer to GFP10 occurred in the NY-β2AR/Gs multiplexed configuration (Ratios between 0.99 and 1.04(f)). However, the transfer of energy between the Rluc attached to βarr-2 (GFP10-linker-RlucII-pβarr2) and the YFP of the NY-β2AR sensor contributed to the BRET signal detected for the βarr2 recruitment to the NY-β2AR in the multiplexed configuration in the presence of ISO (ratio of 1.28) as would be expected from the presence of the NYβ2AR and GFP10-linker-RlucII-pβarr2 in the same complex upon stimulation with ISO. This explains the higher βarr2 engagement response observed with NY-β2AR compared to WT β2AR.

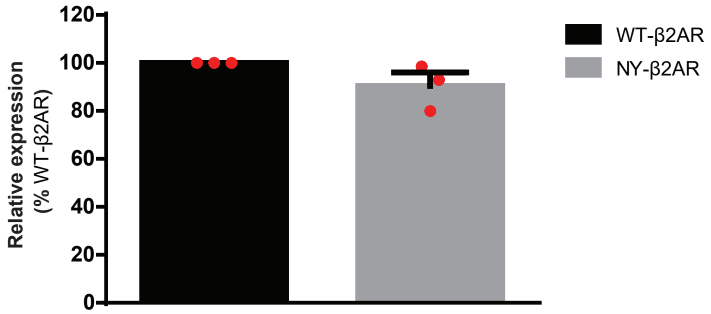

Supplementary Figure 2. Cell surface expression levels of Flag-tagged NY-β2AR conformational sensor and WT β2AR assessed by ELISA. Data are expressed as a % of the expression level of the WT-β2AR. Data represent the mean ± SEM of 3 independent experiments conducted in duplicates. The red dots represent the individual data points obtained in each of the independent experiments. The results show similar levels of receptor on cells expressing either construct.

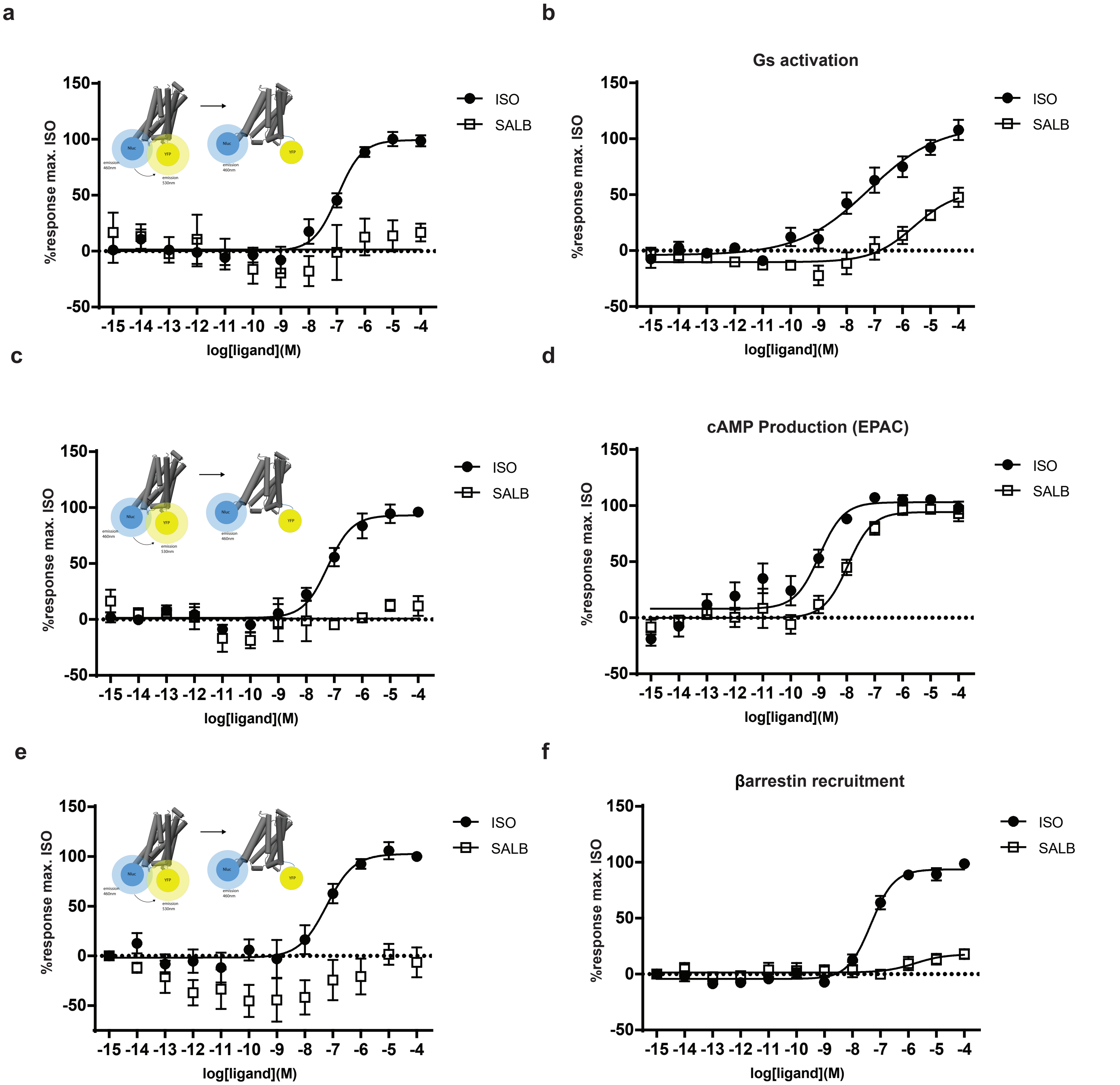

Supplementary Figure 3. Parallel (multiplexed) detection of the conformational biosensor signals and downstream transducer responses. Concentration-response curves were obtained for isoproterenol (ISO) and salbutamol (SALB) in cells co-expressing NY-β2AR and the individual downstream transducer sensors. Ligand-promoted NY-β2AR responses (a-c-e) alongside Gs activation (bimolecular G protein activity sensor Gαs117RlucII/Gγ1-GFP10; b), cAMP production (unimolecular cAMP detecting sensor GFP10-mutEPAC1-RlucII; d) and β-arrestin engagement (unimolecular GFP10-linker-RlucII-pβarr2; f). The signal of the conformational sensor was monitored after addition of coelenterazine 400a and detected using 485 nm (donor)/535 nm (acceptor) emission filters, whereas Gs, EPAC and βarrestin sensor signals were monitored after the addition of methoxy-e-coelenterazine using 400 nm (donor)/510 nm (acceptor) emission filters. Data represent the mean ± SEM of 4 independent experiments conducted in duplicates.

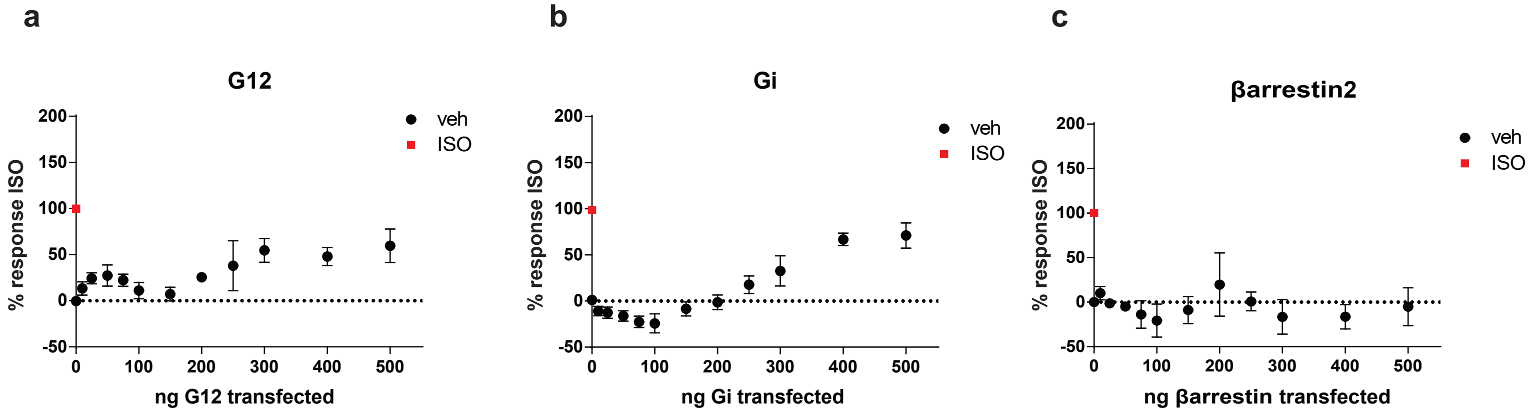

Supplementary Figure 4. Different G protein induced distinct conformational changes in NY-β2AR. G protein-promoted changes in the NY-β2AR conformational sensor BRET signal, as a function of the amount of co-transfected Gα12 (a) , Gαi2 (b) and βarrestin2 (c). Data are expressed as normalized G protein-promoted BRET changes, the maximal response of the biosensor in response to ISO in the absence of over-expressed G protein being set as 100%. Data represent the mean ± SEM of 3 independent experiments conducted in duplicates.

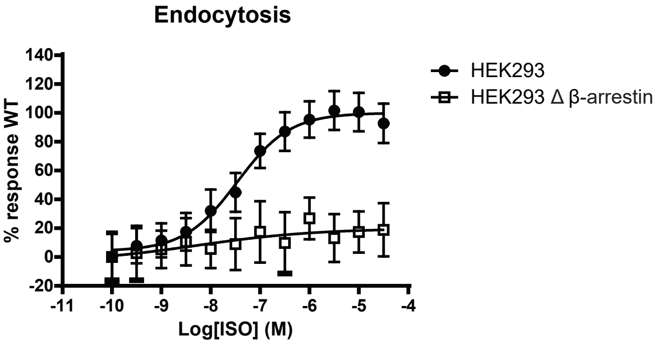

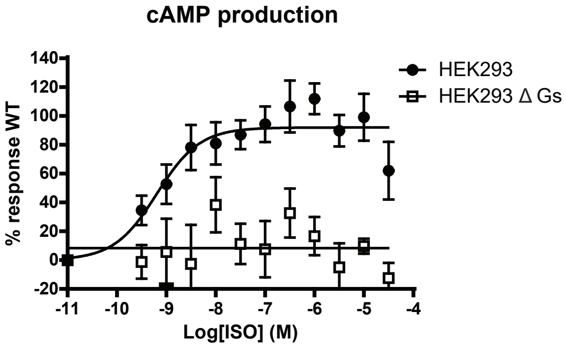

Supplementary Figure 5. Functional characterization of cells in which Gs or βarrestin were inactivated using the CRISPR/Cas9 gene editing system. a) Endocytosis of the β2AR in parental cells and β-arrestin-deficient cells. b) cAMP production by the β2AR upon ISO stimulation in parental cells and Gs-deficient cells. Data are represented as the mean ± SEM of 3 independent experiments. The results confirm the inactivation of Gs and β-arrestin.

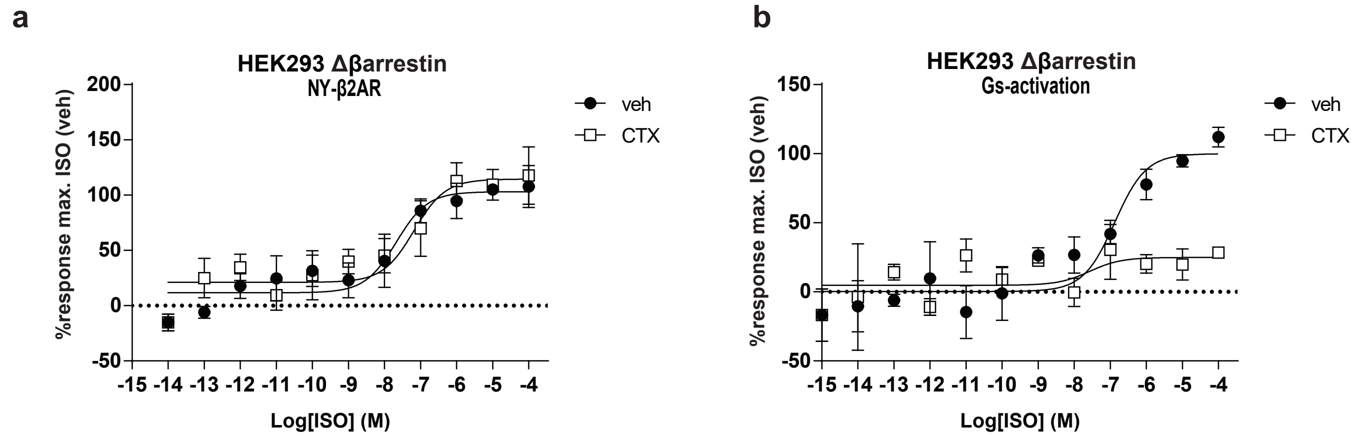

Supplementary Figure 6. ISO-promoted NY-β2AR BRET signal changes in the absence of funtional Gs and β-arrestin. Concentration-response curves of isoproterenol (ISO)-stimulated NY-β2AR expressed in HEK293 cells lacking β-arrestin and pre-treated with cholera toxin (CTX) (200 ng/mL for 18 h) to remove the influence of Gs (a). To control the inactivation of Gs by the CTX treatment, the receptor promoted Gs activation was assessed in the same cell background using the bimolecular G protein activity sensor Gαs117RlucII/Gγ1-GFP10, in the presence and absence of CTX treatment (b). Data are expressed as % response of untreated cells (no CTX). Data represent the mean ± SEM of 3-4 independent experiments conducted in duplicates and indicate that ISO can induce conformational changes independently of Gs and β-arrestin engagement.

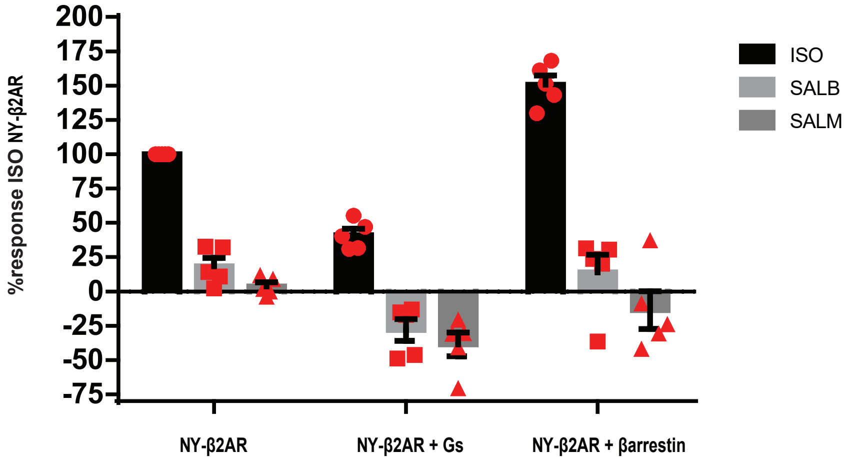

Supplementary Figure 7. Effect of Gs and β-arrestin overexpression on the NY-β2AR conformational sensor response upon stimulation with SALB and SALM. Conformational changes in the NY-β2AR sensor upon stimulation with a saturating concentration (10 μM) of ISO, SALB and SALM. Data are expressed as percentage of ISO-promoted response in the absence of Gs and β-arrestin overexpression and represent the mean ± SEM of 5 independent experiments conducted in duplicates. Results demonstrate that SALM and SALB do not induce detectable conformational changes in cells overexpressing β-arrestin. However, in the presence of overexpressed Gs, both ligands reduce the Gs-promoted response of the NY-β2AR sensor, suggesting stabilization of a similar receptor conformational ensemble by the two ligands for the Gs-bound receptor.
